# Supplementary material for: A Humanized Yeast Phenomic Model of Deoxycytidine Kinase to Predict Genetic Buffering of Nucleoside Analog Cytotoxicity
Source: Genes (Basel). 2019 Sep 30;10(10):770. doi: 10.3390/genes10100770 (PMC6826991; doi:10.3390/genes10100770)
Supplement: Supplementary file 1 [file genes-10-00770-s001.zip › Additional_Files/Additional_File7_GOTermHeatmaps.zip!/Additional_File7_GOTermHeatmaps/nuclear_division.pdf]

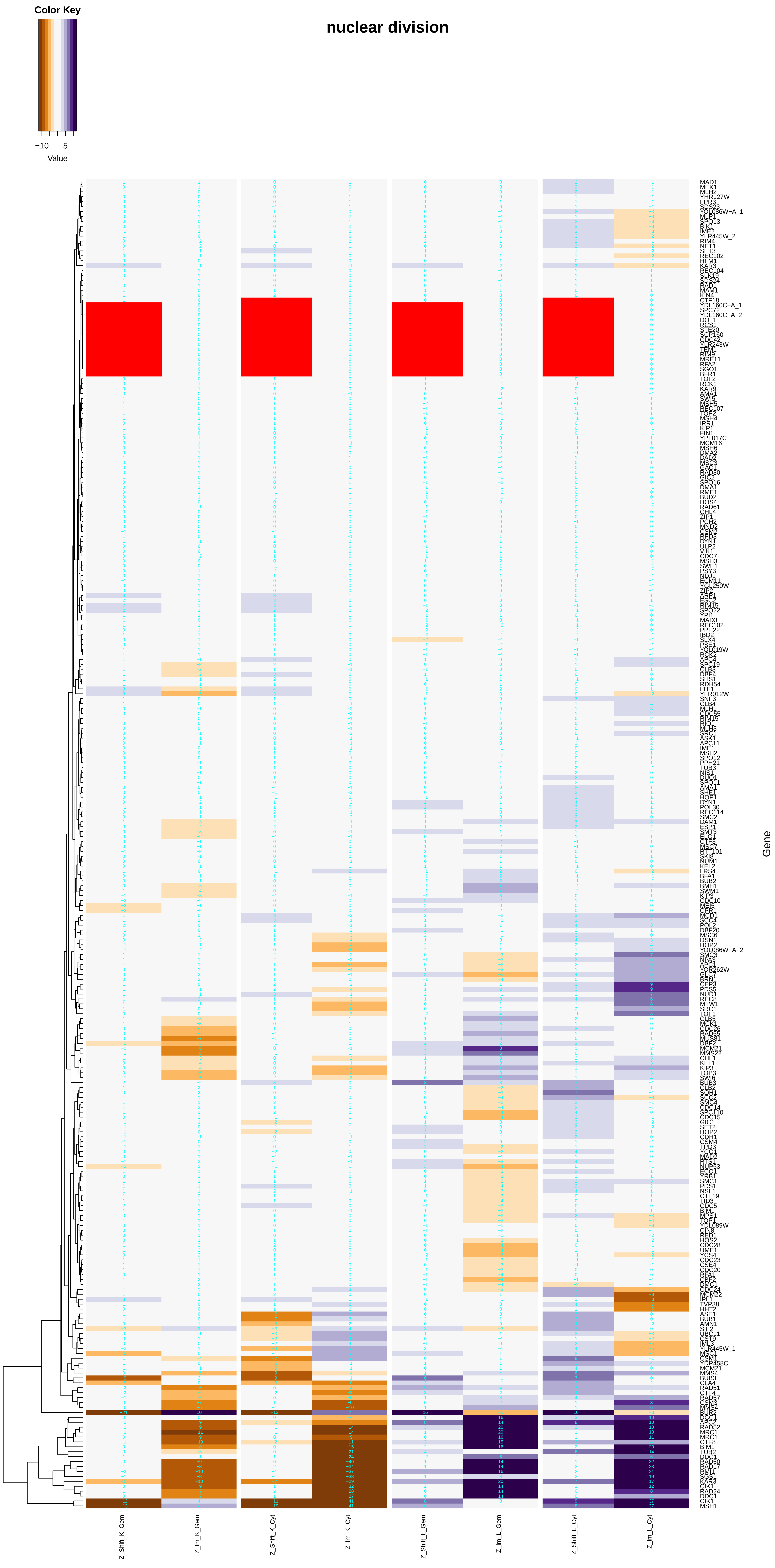
Color Key
−10
5
Value
nuclear division
Gene
Z_Shift_K_Gem
Z_Im_K_Gem
Z_Shift_K_Cyt
Z_Im_K_Cyt
Z_Shift_L_Gem
Z_Im_L_Gem
Z_Shift_L_Cyt
Z_Im_L_Cyt

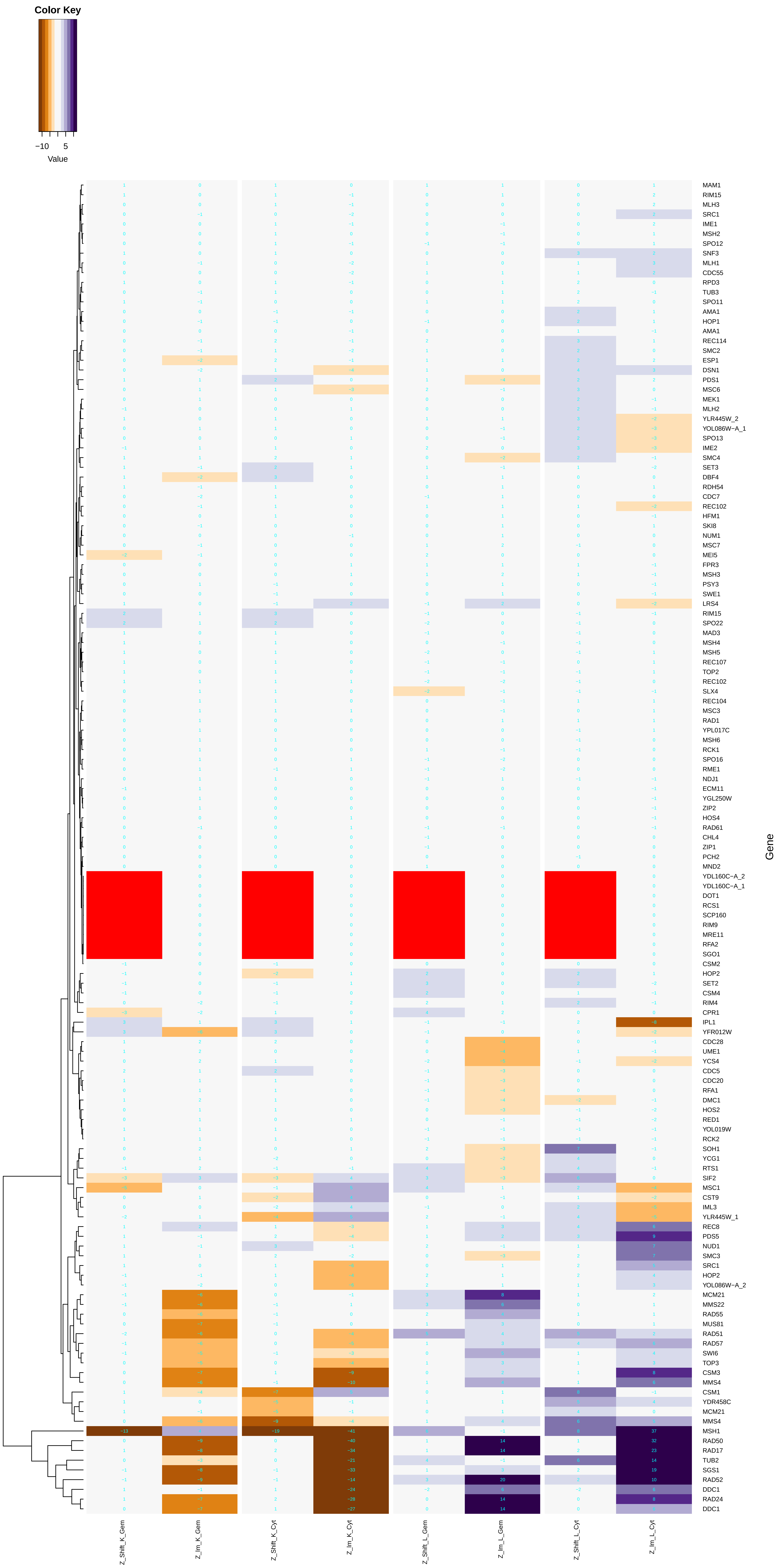
Color Key
−10
5
Value
MAM1
RIM15
MLH3
SRC1
IME1
MSH2
SPO12
SNF3
MLH1
CDC55
RPD3
TUB3
SPO11
AMA1
HOP1
AMA1
REC114
SMC2
ESP1
DSN1
PDS1
MSC6
MEK1
MLH2
YLR445W_2
YOL086W−A_1
SPO13
IME2
SMC4
SET3
DBF4
RDH54
CDC7
REC102
HFM1
SKI8
NUM1
MSC7
MEI5
FPR3
MSH3
PSY3
SWE1
LRS4
RIM15
SPO22
MAD3
MSH4
MSH5
REC107
TOP2
REC102
SLX4
REC104
MSC3
RAD1
YPL017C
MSH6
RCK1
SPO16
RME1
NDJ1
ECM11
YGL250W
ZIP2
HOS4
RAD61
CHL4
ZIP1
PCH2
MND2
YDL160C−A_2
YDL160C−A_1
DOT1
RCS1
SCP160
RIM9
MRE11
RFA2
SGO1
CSM2
HOP2
SET2
CSM4
RIM4
CPR1
IPL1
YFR012W
CDC28
UME1
YCS4
CDC5
CDC20
RFA1
DMC1
HOS2
RED1
YOL019W
RCK2
SOH1
YCG1
RTS1
SIF2
MSC1
CST9
IML3
YLR445W_1
REC8
PDS5
NUD1
SMC3
SRC1
HOP2
YOL086W−A_2
MCM21
MMS22
RAD55
MUS81
RAD51
RAD57
SWI6
TOP3
CSM3
MMS4
CSM1
YDR458C
MCM21
MMS4
MSH1
RAD50
RAD17
TUB2
SGS1
RAD52
DDC1
RAD24
DDC1
Gene
Z_Shift_K_Gem
Z_lm_K_Gem
Z_Shift_K_Cyt
Z_lm_K_Cyt
Z_Shift_L_Gem
Z_lm_L_Gem
Z_Shift_L_Cyt
Z_lm_L_Cyt

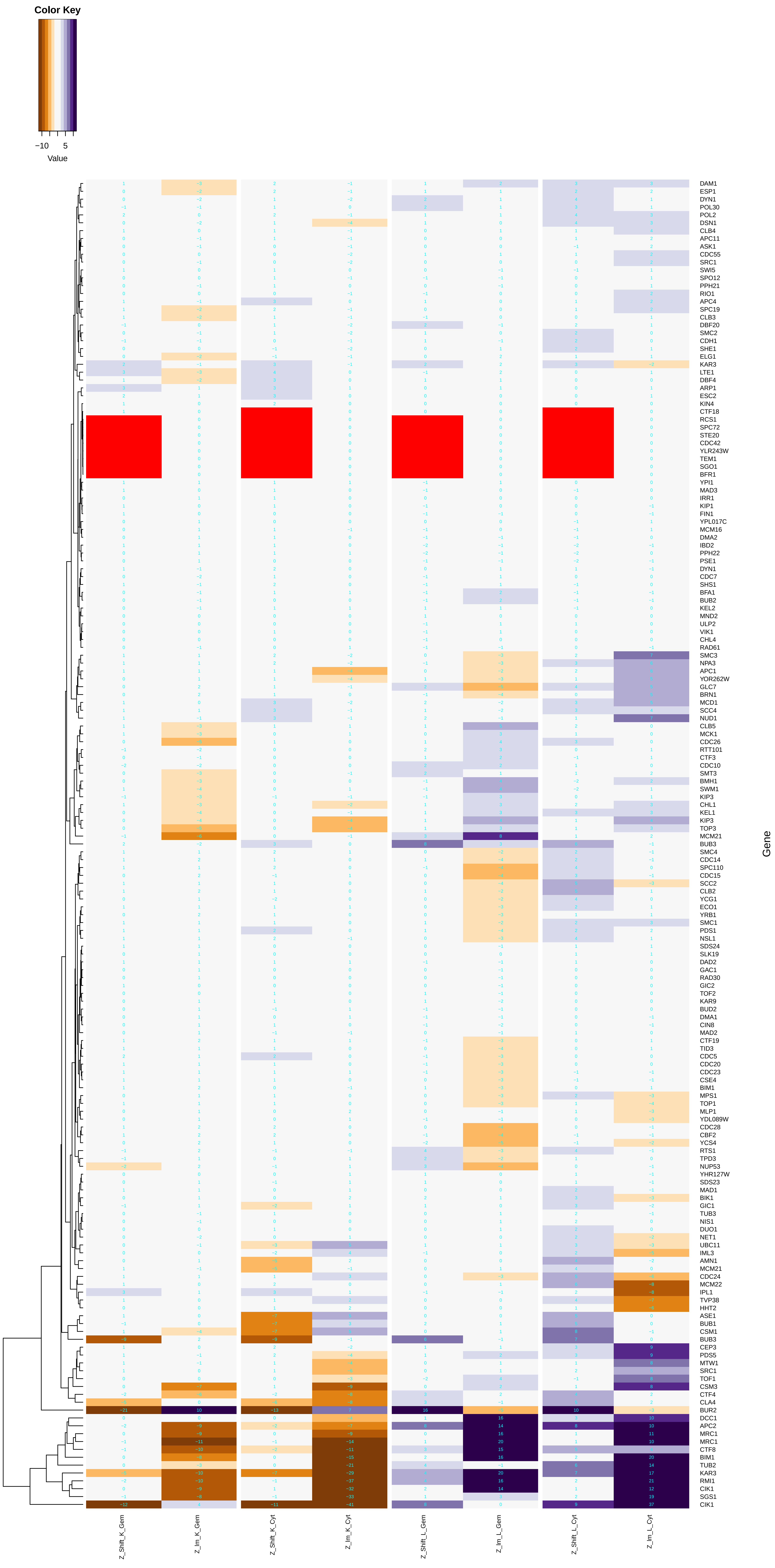
Color Key
−10
5
Value
Gene
DAM1
ESP1
DYN1
POL30
POL2
DSN1
CLB4
APC11
ASK1
CDC55
SRC1
SWI5
SPO12
PPH21
RIO1
APC4
SPC19
CLB3
DBF20
SMC2
CDH1
SHE1
ELG1
KAR3
LTE1
DBF4
ARP1
ESC2
KIN4
CTF18
RCS1
SPC72
STE20
CDC42
YLR243W
TEM1
SGO1
BFR1
YPI1
MAD3
IRR1
KIP1
FIN1
YPL017C
MCM16
DMA2
IBD2
PPH22
PSE1
DYN1
CDC7
SHS1
BFA1
BUB2
KEL2
MND2
ULP2
VIK1
CHL4
RAD61
SMC3
NPA3
APC1
YOR262W
GLC7
BRN1
MCD1
SCC4
NUD1
CLB5
MCK1
CDC26
RTT101
CTF3
CDC10
SMT3
BMH1
SWM1
KIP3
CHL1
KEL1
KIP3
TOP3
MCM21
BUB3
SMC4
CDC14
SPC110
CDC15
SCC2
CLB2
YCG1
ECO1
YRB1
SMC1
PDS1
NSL1
SDS24
SLK19
DAD2
GAC1
RAD30
GIC2
TOF2
KAR9
BUD2
DMA1
CIN8
MAD2
CTF19
TID3
CDC5
CDC20
CDC23
CSE4
BIM1
MPS1
TOP1
MLP1
YDL089W
CDC28
CBF2
YCS4
RTS1
TPD3
NUP53
YHR127W
SDS23
MAD1
BIK1
GIC1
TUB3
NIS1
DUO1
NET1
UBC11
IML3
AMN1
MCM21
CDC24
MCM22
IPL1
TVP38
HHT2
ASE1
BUB1
CSM1
BUB3
CEP3
PDS5
MTW1
SRC1
TOF1
CSM3
CTF4
CLA4
BUR2
DCC1
APC2
MRC1
MRC1
CTF8
BIM1
TUB2
KAR3
RMI1
CIK1
SGS1
CIK1
Z_Shift_K_Gem
Z_Im_K_Gem
Z_Shift_K_Cyt
Z_Im_K_Cyt
Z_Shift_L_Gem
Z_Im_L_Gem
Z_Shift_L_Cyt
Z_Im_L_Cyt

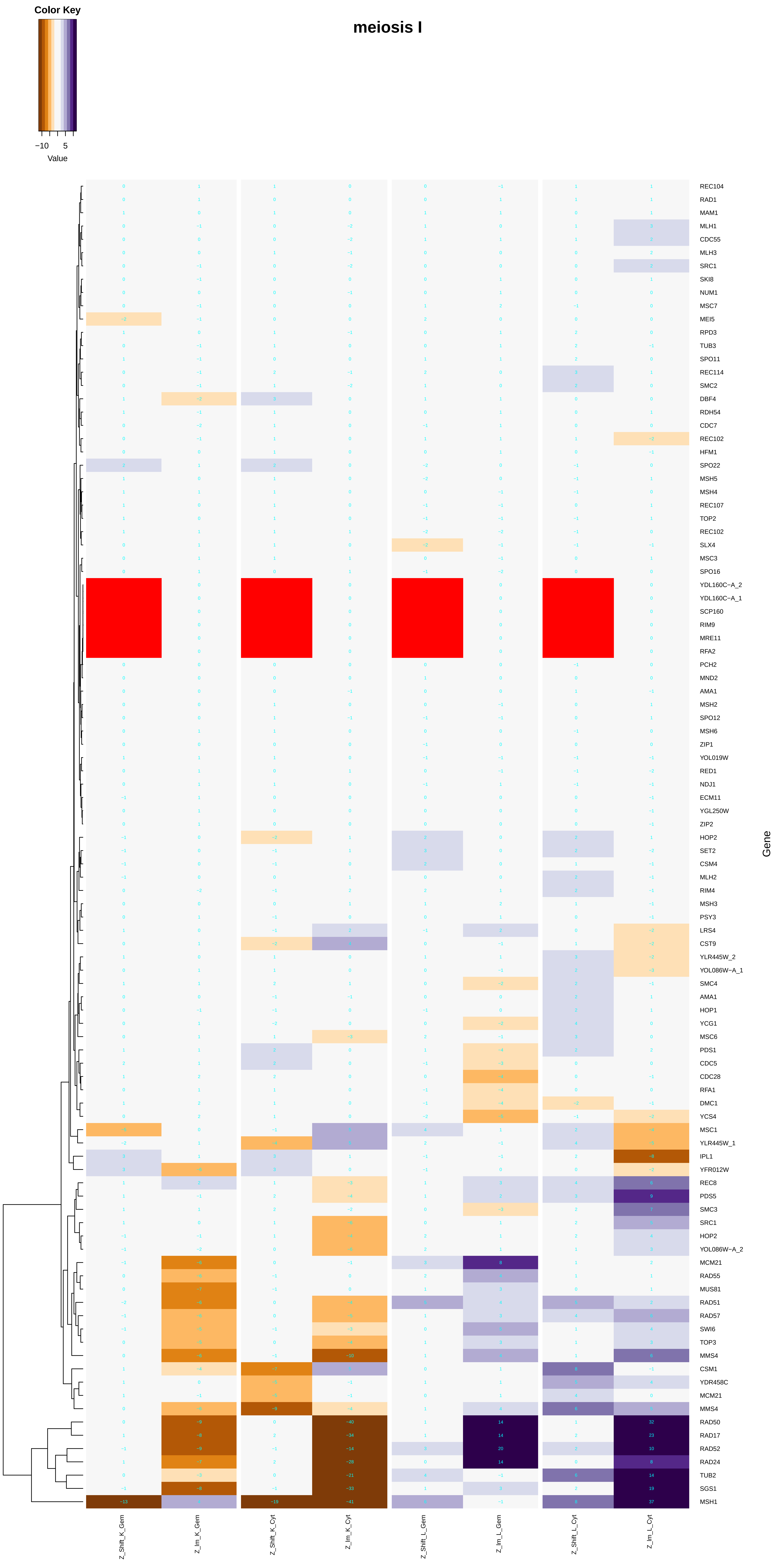
Color Key
−10
5
Value
meiosis I
REC104
RAD1
MAM1
MLH1
CDC55
MLH3
SRC1
SKI8
NUM1
MSC7
MEI5
RPD3
TUB3
SPO11
REC114
SMC2
DBF4
RDH54
CDC7
REC102
HFM1
SPO22
MSH5
MSH4
REC107
TOP2
REC102
SLX4
MSC3
SPO16
YDL160C−A_2
YDL160C−A_1
SCP160
RIM9
MRE11
RFA2
PCH2
MND2
AMA1
MSH2
SPO12
MSH6
ZIP1
YOL019W
RED1
NDJ1
ECM11
YGL250W
ZIP2
HOP2
SET2
CSM4
MLH2
RIM4
MSH3
PSY3
LRS4
CST9
YLR445W_2
YOL086W−A_1
SMC4
AMA1
HOP1
YCG1
MSC6
PDS1
CDC5
CDC28
RFA1
DMC1
YCS4
MSC1
YLR445W_1
IPL1
YFR012W
REC8
PDS5
SMC3
SRC1
HOP2
YOL086W−A_2
MCM21
RAD55
MUS81
RAD51
RAD57
SWI6
TOP3
MMS4
CSM1
YDR458C
MCM21
MMS4
RAD50
RAD17
RAD52
RAD24
TUB2
SGS1
MSH1
Gene
Z_Shift_K_Gem
Z_Im_K_Gem
Z_Shift_K_Cyt
Z_Im_K_Cyt
Z_Shift_L_Gem
Z_Im_L_Gem
Z_Shift_L_Cyt
Z_Im_L_Cyt

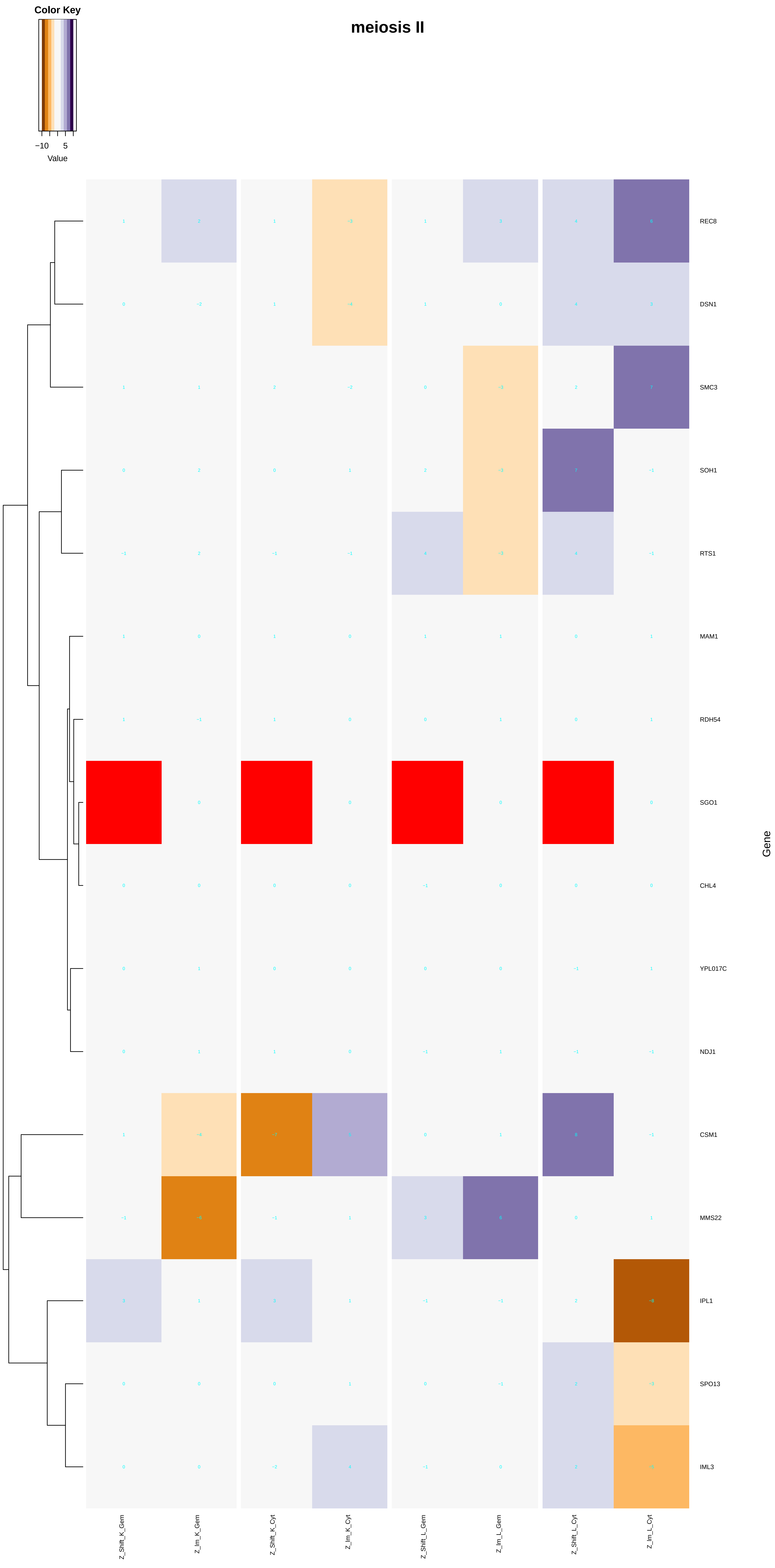
meiosis II
Color Key
−10
5
Value
REC8
DSN1
SMC3
SOH1
RTS1
MAM1
RDH54
SGO1
CHL4
YPL017C
NDJ1
CSM1
MMS22
IPL1
SPO13
IML3
Gene
Z_Shift_K_Gem
Z_Im_K_Gem
Z_Shift_K_Cyt
Z_Im_K_Cyt
Z_Shift_L_Gem
Z_Im_L_Gem
Z_Shift_L_Cyt
Z_Im_L_Cyt
